# Post-intracerebral hemorrhage depression comorbid with obstructive sleep apnea and REM sleep behavior disorder: a case report and literature review

**DOI:** 10.3389/fpsyt.2026.1854271

**Published:** 2026-06-30

**Authors:** Panpan Li, Bidan Zhang, Huihua Li, Jingjing Sun

**Affiliations:** Zhenjiang Mental Health Center, Zhenjiang, Jiangsu, China

**Keywords:** case report, comorbidity, obstructive sleep apnea hypopnea syndrome, post-stroke depression, REM sleep behavior disorder

## Abstract

We report a 63-year-old male with post-cerebral hemorrhage depressive disorder. Previous adequate-dose, full-course antidepressant treatment yielded no significant improvement in depression, anxiety, or sleep disturbance. Detailed history taking and polysomnography (PSG) confirmed comorbid severe obstructive sleep apnea hypopnea syndrome (OSAHS) and rapid eye movement sleep behavior disorder (RBD). Without adjusting antidepressants, the patient first received continuous positive airway pressure (CPAP) for OSAHS, followed by individualized pharmacotherapy for RBD. With the improvement of sleep architecture and respiratory events, obvious remission of depressive and anxiety symptoms and substantial recovery of daytime function were observed. This case emphasizes the bidirectional relationship between sleep and mood disorders. For post-stroke depression (PSD) poorly responsive to antidepressants, active sleep evaluation, multidisciplinary collaboration, and targeted management of comorbid sleep disorders may be crucial to enhance therapeutic efficacy.

## Introduction

Post-stroke depression (PSD) is one of the common and severe sequelae following stroke, with a prevalence ranging from 31.3% to 41.5% (1), and most depressive episodes occur within 3 months after stroke (2). PSD is often complicated by sleep disorders, with the two conditions mutually exacerbating each other’s severity (3). Among sleep-related comorbidities, obstructive sleep apnea hypopnea syndrome (OSAHS) and rapid eye movement sleep behavior disorder (RBD) are particularly noteworthy in patients with PSD (4–6). This case report describes an instance of post-intracerebral hemorrhage depression complicated by OSAHS and RBD, highlighting the clinical importance of identifying and intervening in coexisting sleep disorders in patients with PSD. Meanwhile, this case offers clinical reference that combined intervention on sleep comorbidities helps optimize treatment effects for PSD associated with cerebrovascular diseases.

## Report of case

A 63-year-old male patient sustained a cerebral hemorrhage on September 28, 2024. Cranial CT revealed intracerebral hemorrhage in the left thalamus with ventricular rupture and age-related cerebral atrophy. Residual dysarthria and unsteady gait were noted after neurological treatment. Two months later, he gradually developed depressed mood, diminished interest in routine activities, irritability, occasional restlessness and excessive anxiety about poor disease recovery. He also experienced sleep disturbance, frequent dreaming, easy awakening and memory decline. He was previously diagnosed with organic mental disorder in the outpatient department of Zhenjiang Mental Health Center, and was administered sertraline 200 mg once daily, idebenone 30 mg three times daily, and intermittent zolpidem tartrate 10 mg once daily at bedtime. However, his mood and interest showed no significant improvement after medication, and he still complained of persistent fatigue, daytime sleepiness, dizziness, poor nighttime sleep quality and frequent dreaming. He was thus admitted to the Department of Sleep Medicine, Zhenjiang Mental Health Center on October 29, 2025 for further treatment.

He had a 10-year history of hypertension, managed with nifedipine sustained-release and labetalol hydrochloride tablets, and his blood pressure was well controlled. He also had a history of cerebral hemorrhage and intracranial aneurysm stenting, and took aspirin enteric-coated tablets and clopidogrel bisulfate tablets for antiplatelet therapy.

Further medical history inquiry revealed that the patient had suffered from frequent nightmares for more than 10 years. The nightmares mainly featured quarrels, fights and terrifying scenes. Meanwhile, he presented motor behaviors including loud shouting, punching and kicking during sleep, and he had even fallen out of bed on several occasions. There was no family history of similar diseases. The above symptoms worsened significantly after the cerebral hemorrhage. He also had obvious, loud and irregular snoring during sleep, accompanied by excessive daytime sleepiness.

Physical Examination: BMI 22.0 kg/m²; clear consciousness; unsteady gait; muscle strength grade 5 in both left and right limbs with normal muscle tension.

Psychiatric Examination: Clear consciousness and intact orientation. The patient presented with a slightly distressed appearance, depressed mood, anhedonia, fatigue and worry about his own illness. He denied suicidal ideation, and no psychotic symptoms such as hallucinations or delusions were elicited. Sleep disturbances included prolonged sleep onset latency, recurrent violent nightmares accompanied by vocalization and limb movements, as well as loud irregular snoring; He had mild recent memory impairment, while remote memory and insight remained intact.

Auxiliary Examinations: Routine blood, urine and stool tests, blood biochemistry, thyroid function, coagulation profile, hepatitis B serology, and four infectious disease markers showed no abnormalities. The electrocardiogram was normal, and the electroencephalogram showed mild abnormalities. Chest CT indicated a small nodule in the right lower lobe of the lung. Cranial magnetic resonance imaging on July 1, 2025 demonstrated patchy hypointensities in the left thalamus and basal ganglia, consistent with chronic changes of resorbed hemorrhage, age-related cerebral atrophy, and an aneurysm in the C6 segment of the right internal carotid artery.

Scale Assessment: HAMD: 17 points (mild depression); HAMA: 15 points (definite anxiety); ISI: 21 points (moderate insomnia).

Polysomnography (PSG): The patient was monitored overnight with the Compumedics Grael PSG system. A technologist remained on site throughout the study. Sleep architecture and relevant events were manually scored following the American Academy of Sleep Medicine (AASM) Manual for the Scoring of Sleep and Associated Events, Version 3.0. Both nights of PSG revealed elevated electromyographic activity during REM sleep. PSG video revealed abnormal movements such as hand waving in the patient during REM sleep. Detailed values are presented in **Table 1**.

**Table 1:** Results of two PSG recordings during hospitalization.

Main sleep parameters	First PSG result (2025/10/29)	Second PSG result (2025/10/30)	Normal reference range
SL(min)	21.0	9.5	10-30
REML(min)	4.5	42.5	60-120
N1(%)	40.5	40.6	5–10
N2(%)	29.4	43.3	50–60
N3(%)	0	0	15–20
REM(%)	30.1	16.0	20–25
wakefulness after sleep onset(min)	113.5	128.0	<30
TST(min)	338.5	349.5	420-480
Sleep Efficiency(%)	71.6	71.8	>85
AHI(events/h)	42.4	31.8	<5
PLMS index (events/h)	10.3	33.0	<5
Min SpO_2_(%)	86	84	>90

SL, Sleep Latency; REML, REM sleep latency; N1, NREM stage 1 sleep; N2, NREM stage 2 sleep; N3, NREM stage 3 sleep; TST, total sleep time; AHI, apnea-hypopnea index; PLMS index, Periodic Limb Movements During Sleep Index; Min SpO_2_, Minimum oxygen saturation.

### Diagnoses

1. Post-stroke depression.
2. Severe obstructive sleep apnea hypopnea syndrome.
3. Rapid eye movement sleep behavior disorder.
4. Hypertension.
5. Sequelae of cerebral hemorrhage.
6. Post intracranial aneurysm stent implantation.

Treatment Course: The treatment process is summarized in **Table 2**. The medication summary reflects treatment outcomes and guides individualized drug selection for similar cases.

**Table 2:** Patient’s treatment course.

Treatment stage	OSAHS	RBD	Insomnia	Depression
Week 1	CPAP	–	CBT-I	sertraline 200 mg
Week 2	CPAP	clonazepam 0.5 mg	CBT-I	sertraline 200 mg
Week 3	CPAP	clonazepam 0.25 mg	CBT-I	sertraline 200 mg
Week 4	CPAP	clonazepam 0.25 mg	CBT-I	sertraline 200 mg
1-month follow-up	CPAP	clonazepam 0.25 mg	CBT-I	sertraline 200 mg

In the first week, continuous positive airway pressure (CPAP) therapy was initiated for OSAHS. A ResMed AirCurve 10 bilevel sleep ventilator with an N5 mask was used for auto-titrating mode. The apnea-hypopnea index (AHI) decreased from 31.8 events per hour to approximately 6 events per hour. Cognitive behavioral therapy for insomnia (CBT-I) was provided by a professional psychotherapist. The therapy was delivered once weekly for 50 minutes per session, covering sleep hygiene guidance, stimulus control therapy, sleep restriction therapy, cognitive restructuring, relaxation training, and sleep diary review. Sertraline 200 mg daily was maintained.

In the second week, the patient gradually adapted to non-invasive positive pressure ventilation. Treatment adherence was monitored using the built-in recorder of the CPAP device. Data demonstrated nightly usage of 6.5 to 8 hours, and the AHI remained below 5 events per hour, reflecting satisfactory adherence. The patient reported improved sleep quality. Clonazepam 0.5 mg was administered at bedtime for RBD, and other therapies were unchanged.

In the third week, the patient’s mood improved gradually, accompanied by decreased nocturnal shouting and marked relief of dream-associated motor activities. Mild lower limb weakness was noted thereafter. Given this adverse reaction, the clonazepam dose was tapered to 0.25 mg, and other treatments were continued as before.

In the fourth week, CPAP, CBT-I, sertraline 200 mg daily and clonazepam 0.25 mg at bedtime were maintained. No residual lower limb weakness was detected. Overall sleep status improved remarkably: the ISI score dropped to 3, the AHI was stabilized at approximately 5 events per hour, sleep diary data showed satisfactory sleep with sleep efficiency above 85%, and excessive daytime sleepiness was alleviated. The patient was subsequently discharged.

Without antidepressant adjustment, the HAMD score decreased from 17 to 2 points and the HAMA score from 15 to 5 points, indicating marked remission of depression and anxiety. Dreaming was reduced, and shouting and limb movements almost disappeared. At the one-month follow-up after discharge, the patient continued to take sertraline 200 mg daily, clonazepam 0.25 mg nightly and CPAP. The AHI remained controlled at approximately 5 events per hour, his mood was stable, and nocturnal shouting and limb movements were basically controlled.

We have compiled the patient’s disease progression timeline in **Table 3**. The timeline illustrates the clinical course of the disease, revealing the value of longitudinal symptom observation.

**Table 3:** Timeline diagram of the clinical course.

Time point	Event/clinical details
10 years ago (approximately)	Loud and irregular snoring during sleep. Frequent nightmares during sleep, accompanied by loud shouting, punching and kicking.
Sep, 2024	Cerebral hemorrhage occurred. Cranial CT revealed intracerebral hemorrhage in the left thalamus with ventricular rupture, age-related cerebral atrophy.
Dec 2024	Progressive depressed mood, anhedonia and sleep disturbance
Dec, 2024	Diagnosed with organic mental disorder at Zhenjiang Mental Health Center (Outpatient). Treated with sertraline, titrated gradually to 200 mg.
July, 2025	Cranial magnetic resonance imaging demonstrated patchy hypointensities in the left thalamus and basal ganglia, consistent with chronic changes of resorbed hemorrhage, age-related cerebral atrophy, and an aneurysm in the C6 segment of the right internal carotid artery.
Oct 21, 2025	intracranial aneurysm stenting
Oct 29, 2025	Admitted to Department of Sleep Medicine, Zhenjiang Mental Health Center
Oct 29, 2025	First PSG examination
Oct 30, 2025	Second PSG examination

## Discussion

PSD is one of the most common and severe complications following stroke, affecting approximately one-third of stroke survivors (7). Its primary clinical manifestations include depressed mood, anhedonia, and fatigue. Compared with depressed patients in the general population, individuals with PSD experience more frequent and severe emotional dysregulation, including catastrophic reactions, emotional lability and diurnal mood fluctuations, along with markedly impaired occupational function (8). Additionally, PSD is significantly associated with a higher mortality rate (7, 9). The pathogenesis of PSD remains incompletely understood, with proposed mechanisms including lesions and disrupted neural networks in the brain, hypothalamic-pituitary-adrenal (HPA) axis dysregulation, increased inflammatory factors, reduced monoamine neurotransmission, glutamate-mediated excitotoxicity, abnormal neurotrophic responses, white matter hyperintensities, gut microbiota-gut-brain axis dysfunction, epigenetic modifications, decreased brain-derived neurotrophic factor expression, and psychosocial factors (7, 9–15). Nevertheless, in clinical practice, some patients with PSD fail to achieve adequate symptomatic improvement even after standard antidepressant treatment. In such cases, apart from stroke-induced neurobiological changes, clinicians should pay close attention to undetected comorbidities like sleep disorders.

In this case, the patient presented typical depressive symptoms following cerebral hemorrhage, but showed minimal mood improvement despite standardized antidepressant treatment, with persistent poor sleep quality, daytime sleepiness, and fatigue. The patient had experienced these symptoms for a prolonged period before referral for sleep medicine evaluation. This finding suggests that sleep-related comorbidities are often detected late in routine clinical practice. Further history taking revealed chronic snoring, nocturnal awakenings, and daytime somnolence, and subsequent PSG confirmed severe OSAHS. Additionally, the patient had a long history of dream-associated shouting and limb movements; PSG demonstrated REM sleep without atonia with elevated electromyographic activity during REM sleep, consistent with a diagnosis of RBD. This case indicates that for PSD patients who show a poor respond to antidepressant drugs andpersist with severe sleep disturbances, clinicians should obtain a detailed sleep history. Screening for common sleep disorders such as sleep apnea and abnormal nocturnal behaviors prevalent among middle-aged and elderly men is recommended. Timely PSG detection is also essential, as it avoids the misjudgment of comorbid sleep symptoms as insufficient therapeutic responses to primary cerebrovascular disease.

It is noteworthy that this patient had concurrent PSD, OSAHS and RBD. Depressive symptoms achieved obvious improvement after targeted intervention for sleep disorders, while the dosage of antidepressants remained unchanged. The clinical observation indicates a potential link between relieved sleep disturbances and improved depressive state. Further research is still required to clarify the interplay of factors affecting the development of depression. OSAHS is characterized by recurrent collapse and obstruction of the upper airway during sleep, resulting in periodic hypopnea or apnea, followed by hypoxemia, hypercapnia, and sleep fragmentation (16–18). Patients with OSAHS frequently exhibit comorbid depression and anxiety (19). The prevalence of depression in OSAHS is significantly higher than in the general population (20), and OSAHS constitutes an independent prospective risk factor for depression (21). Multiple large-scale studies have confirmed (22–24) that even undiagnosed or high-risk OSAHS independently increases the risk of depression, anxiety, and even suicidal behavior in middle-aged and elderly individuals. Underlying mechanisms include intermittent hypoxia, disrupted sleep architecture, autonomic and HPA axis dysregulation, neuroinflammation, abnormal brain function, and altered expression of circadian clock genes (e.g., PER1) (20, 25). Studies have confirmed a long-term independent dose-response relationship between the severity of sleep-disordered breathing and depression. Higher disease grades and AHI correspond to an increased risk of developing depression (26, 27). OSAHS significantly increases the risk of stroke (4). It is not only an independent risk factor for stroke but is also closely associated with poor stroke prognosis, increased risk of recurrence, and higher mortality (28). The most commonly reported sleep disorder after stroke is OSAHS (29), and stroke may induce or exacerbate OSAHS through mechanisms such as impaired upper airway muscle tone, altered ventilatory control, and changes in sleep structure and posture after stroke (30). Previous studies have shown that the severity of OSAHS in patients with ischemic stroke is positively correlated with PSD three months after onset. The intermittent nocturnal hypoxia caused by OSAHS can trigger oxidative stress and systemic inflammation, worsen cerebral small vessel damage, and disrupt the balance of central neurotransmitters, thereby increasing the risk of developing PSD (31). Besides, patients with OSAHS commonly experience fatigue, poor concentration and low mood. These symptoms overlap with some manifestations of PSD, so they are easily mistaken for persistent depressive symptoms alone, which hinders timely identification and targeted intervention. Timely and effective CPAP therapy in OSAHS patients significantly improves depressive symptoms and neurocognitive function (32), with greater benefits observed in those with more severe baseline depression (20). Higher adherence to PAP therapy is associated with lower rates of self-harm incidents and reduced healthcare utilization (33). Conversely, untreated OSAHS exacerbates affective disorders, frequently causes cognitive decline, and may even result in permanent brain injury (34).

RBD (35, 36) is a sleep disorder characterized by the loss of muscle atonia during REM sleep accompanied by abnormal behaviors such as dream enactment. It is classified into idiopathic RBD (iRBD) and secondary RBD. RBD represents a prodromal stage of neurodegenerative diseases including Parkinson’s disease (PD), dementia with Lewy bodies, and multiple system atrophy, with up to 80% of patients eventually developing these conditions. The prevalence of iRBD with dream-enacting behaviors is approximately 1% in individuals aged 60 years and older (35, 37). RBD is associated with an increased risk of stroke (38), and excessive sympathetic activation and dopamine neurotransmitter deficiency may be potential underlying mechanisms (39). The coexistence of OSAHS in RBD patients is very common, with a prevalence of 34%-61% (40). There is a bidirectional interaction between the two: sleep fragmentation and intermittent hypoxemia related to OSAHS may exacerbate RBD symptoms by disrupting REM sleep structure and affecting brainstem function, while REM-related muscle atonia associated with RBD may partially alleviate OSAHS severity during REM periods. This complex interaction may stem from shared risk factors (such as advanced age and male sex), as well as potential shared pathophysiological mechanisms involving brainstem structures responsible for sleep regulation and upper airway control (41). Patients with iRBD have impaired emotional regulation (42), presenting notably more severe depressive symptoms compared with control groups (37, 43, 44). Previous research suggests that age and disease duration are not significantly correlated with depressive symptoms in iRBD patients (43). By contrast, clinical severity (45), episodes of violent or elaborate dream enactment, and the index of dream behaviors linked to negative emotions are positively associated with depression (46). However, a meta-analysis by Sumi et al. (47) demonstrated an inverse relationship between depression severity and disease duration in iRBD, which may be attributable to a reduction in dream enactment behavior, alleviation of psychological stress, and adaptation to the disease. The regulatory mechanisms underlying the association between depression and iRBD remain unclear. Both conditions are prodromal manifestations of α-synucleinopathies, and their comorbidity may be mediated by early pathological processes in neurodegenerative disorders such as PD (48–50). Besides, these conditions share polygenic hereditary susceptibility (51). Dysfunction of the serotonergic system in the dorsal raphe nucleus (52) and disrupted circadian rhythms are likely to contribute to depression among patients with iRBD (43, 53). These individuals also have unstable REM sleep and disrupted transitions between REM and non-REM sleep stages. Such changes may impair the emotional regulation network formed by the amygdala and prefrontal cortex, serving as a key pathological mechanism for the onset of depression (43). Furthermore, the potential impact of antidepressants on RBD symptoms should be considered. The patient received relatively high-dose sertraline for a long time. SSRIs have been proven to induce or exacerbate REM sleep without atonia and relevant RBD manifestations (54–56). Hence, the aggravation of RBD in this case may be attributed to neurological dysfunction after intracerebral hemorrhage, with medication-related factors also potentially involved.

This study has several inherent limitations. As a single-case clinical report, it lacks long-term follow-up records, so the research findings cannot be widely applied to similar patients. The patient received multiple interventions simultaneously, including antidepressant medication, CPAP, sleep behavioral therapy and clonazepam. It is hard to distinguish the individual effect of each treatment brought to symptom relief. Selective serotonin reuptake inhibitors may also interfere with RBD manifestations and bring confounding effects. Another limitation lies in the change of sleep parameters. The PLMS index increased obviously during PSG monitoring. However, the patient did not exhibit clinically predominant restless legs syndrome symptoms. It should be noted that sleep fragmentation related to PLMS may have partially contributed to sleep disruption. Further large-scale prospective trials are required to clarify the interaction and underlying mechanisms between post-stroke depression and sleep disorders.

## Conclusion

In clinical practice, the bidirectional association between sleep and mood disorders should be emphasized. It is not advisable to make diagnosis from a single perspective. Medical staff should actively inquire about patients’ sleep details such as snoring and abnormal nocturnal body movements, and arrange timely PSG examination to reduce missed diagnoses of comorbid conditions. Reinforcement of multidisciplinary teamwork collaboration is imperative. This case initially involved only neurology and psychiatry, missing sleep medicine and leading to unrecognized sleep comorbidities. This case supports early multidisciplinary collaboration. The approach applies to cerebral hemorrhage patients with complicated systemic symptoms or ineffective single therapy. Joint medical work helps formulate full intervention strategies and deliver consistent whole-course treatment. This case encompassed neurological rehabilitation, organic psychiatric disorders, OSAHS, and RBD—beyond single-department management. Through neurology-psychiatry-sleep medicine collaboration, sleep disorders may have contributed substantially to the persistence of psychiatric symptoms. An individualized plan centered on CPAP (for OSAHS) + targeted RBD treatment (clonazepam) with adjunctive psychotropics was implemented, resulting in significant symptom alleviation.

## Data Availability

The original contributions presented in the study are included in the article/supplementary material. Further inquiries can be directed to the corresponding authors.
